# Adverse Psychiatric Effects Associated with Herbal Weight-Loss Products

**DOI:** 10.1155/2015/120679

**Published:** 2015-09-17

**Authors:** F. Saverio Bersani, Marialuce Coviello, Claudio Imperatori, Marta Francesconi, Christina M. Hough, Giuseppe Valeriani, Gianfranco De Stefano, Flaminia Bolzan Mariotti Posocco, Rita Santacroce, Amedeo Minichino, Ornella Corazza

**Affiliations:** ^1^Department of Neurology and Psychiatry, Sapienza University of Rome, 00185 Rome, Italy; ^2^School of Life and Medical Sciences, University of Hertfordshire, Hatfield AL10 9AB, UK; ^3^Department of Human Sciences, European University of Rome, 00163 Rome, Italy; ^4^Department of Psychiatry, University of California San Francisco, San Francisco, CA 94143, USA; ^5^Department of Psychiatry, Sahlgrenska University Hospital, 413 45 Gothenburg, Sweden; ^6^International Association for Applied Human Sciences, 00100 Rome, Italy; ^7^Department of Neuroscience and Imaging, Gabriele D'Annunzio University, 66100 Chieti, Italy

## Abstract

Obesity and overeating are among the most prevalent health concerns worldwide and individuals are increasingly using performance and image-enhancing drugs (PIEDs) as an easy and fast way to control their weight. Among these, herbal weight-loss products (HWLPs) often attract users due to their health claims, assumed safety, easy availability, affordable price, extensive marketing, and the perceived lack of need for professional oversight. Reports suggest that certain HWLPs may lead to onset or exacerbation of psychiatric disturbances. Here we review the available evidence on psychiatric adverse effects of HWLPs due to their intrinsic toxicity and potential for interaction with psychiatric medications.

## 1. Introduction

Novel Psychoactive Substances (NPSs) comprise an ever-increasing number of chemical, pharmaceutical, and herbal drugs that are often advertised as “legal” and “safer” alternatives to International Controlled Drugs (ICDs) [1, 2]. While the use of ICDs seems to have stabilized over the past decades, the market of NPSs has significantly grown, representing an unprecedented challenge in the field of global health from medical, social, cultural, legal, and political perspectives [1–11]. Among NPSs, a rapid spread of substances known as “performance and image-enhancing drugs” (PIEDs) or “lifestyle drugs” has been recorded [12, 13]. The term PIED is an umbrella term used to describe a variety of substances taken to enhance human abilities [14]. More specifically, PIEDs include a wide range of substances able to modify (i) muscles' structure and function; (ii) weight, skin coloration, hairs, aging processes, and body-image in general; (iii) sexual behaviours; (iv) cognitive functioning; and (v) mood and social behaviours [12–14].

Obesity and overeating are becoming one of the most prevalent health concerns among all populations and age groups worldwide, resulting in a significant increase in mortality and morbidity [15, 16]. Though diet, exercise, and healthy lifestyle are the mainstays of obesity management [15, 16], the use of PIEDs is increasing as it is viewed as an easier alternative to controlling weight [17–20]. In addition, the use of PIEDs is also increasing in nonobese and nonovereating populations [12]. The cause of this is most likely modern society's high focus on physical appearance, often prompting individuals to attempt to achieve unrealistic standards of thinness [12, 21].

Among the different PIEDs, herbal weight-loss products (HWLPs) often attract users due to their health claims, assumed safety, easy availability from specialised shops and the Internet, affordable price, extensive marketing, and the perceived lack of need for professional oversight [17–20]. Certain HWLPs may have a significant antiobesity effect [22], with some studies reporting potential somatic adverse effects associated with their use [18, 19, 23]. Associations between the use of HWLPs and onset or exacerbation of psychiatric symptoms, including mood disturbances, anxiety, psychosis, addiction, and delirium have also been reported (reviewed below). There are two main mechanisms through which HWLPs may have detrimental effects on mental health: (i) intrinsic toxicity of the herbs and/or (ii) interactions between herbs and psychiatric medications [18]. However, the relationship between HWLPs and psychiatric disturbances has been relatively unstudied so far, and there is a lack of structured and consistent research on the topic.

The information provided in the studies reporting theoretical associations between HWLPs and psychiatric symptoms is often (i) based on single-case reports and (ii) insufficient for making reliable inferences about causality. Therefore, rather than drawing conclusions, the purpose of this review is to aggregate and report available data, to heighten awareness of the possible relationship between HWLPs and adverse psychiatric effects, and to encourage future studies to include mental health measures in relation to this rapidly growing pattern of substance use.

## 2. Methods

In the following sections, we review the evidence of HWLPs adverse effects relevant to psychiatric conditions. In Section 3 we review the evidence of adverse psychiatric effects of HWLPs that are attributable to the intrinsic toxicity of the herbs; in Section 4 we review the evidence of interactions between HWLPs and psychiatric medications. Our review strategy started with MedLine searches to identify herbal products with weight-loss properties. We then performed more specific MedLine searches to explore adverse psychiatric effects (on humans) and interactions with psychiatric medications for each herbal product with documented weight-loss properties.

## 3. Intrinsic Toxicity of Weight-Loss Herbal Products

### 3.1. *Panax ginseng*

*Panax ginseng* (ginseng) is a perennial herb native to Korea and China, the root of which has been traditionally used as an “adaptogenic” substance, that is, a compound meant to nonspecifically contribute to the stabilization of physiological processes and the promotion of health and homeostasis [24]. It is often used for weight-loss purposes, as it has been suggested to improve glucose tolerance, reduce LDL plasma levels, and contribute to global inhibition of body weight gain [19, 25]. There is evidence of ginseng modulatory activity on the noradrenergic, dopaminergic, serotoninergic, cholinergic, and endorphin systems [26–28], and there have been several reports of psychiatric symptoms associated with the use of ginseng both in healthy people and in patients previously affected by psychiatric disorders (reviewed below).

In a psychological examination of 133 long-term ginseng users, 18 cases of euphoria (after one-week use), 25 cases of nervousness (after one-week use), 26 cases of sleeplessness (after 3-week use), and 6 cases of depression (after 24-week use) have been recorded, with high single doses (15 g) causing feelings of depersonalization and confusion in 4 subjects [29]. Anxiety and/or sleep disturbances were reported among healthy volunteers taking ginseng monopreparations for 12 weeks (200 mg/day) [30] or for 30 days (1000 mg/day) [31] in placebo-controlled trials [30, 31], as well as in healthy individuals taking ginseng combination preparations [24]. In the World Health Organization (WHO) Collaborating Centre for International Drug Monitoring Database and UK Medicines Control Agency reports of adverse events, ginseng monopreparations have been associated with cases of insomnia, nervousness, manic reaction, amnesia, somnolence, anorexia, anxiety, emotional lability, hallucination, sleep disorder, confusion, depression, and abnormal thinking [24].

Some studies have also reported associations in the exacerbation or worsening of psychiatric symptoms with ginseng use in patients affected by psychiatric disorders. Two case reports by Gonzalez-Seijo et al. and Vázquez and Agüera-Ortiz suggest that ginseng may have contributed to inducing a manic episode in patients with previous diagnoses of affective disorders [32, 33]. Gonzalez-Seijo et al. described the case of a woman suffering from recurrent depressive episodes who experienced a manic episode a few days after having self-replaced her therapy (lithium carbonate 1200 mg/day and amitriptyline 75 mg/day) with one tablet of ginseng every day [32]. It is not known, however, if ginseng* per se* may have induced the manic episode or, rather, the manic episode was precipitated by amitriptyline or lithium withdrawal [32]. Vázquez and Agüera-Ortiz reported the case of a 56-year-old woman with previous major depressive disorder with psychotic symptoms who presented a manic episode during ginseng intake [33]; symptoms disappeared rapidly with low doses of neuroleptics and benzodiazepines after ginseng suppression [33]. However, Vázquez and Agüera-Ortiz could not clarify the effective contribution of the ginseng suppression to the amelioration of symptoms [33]. A third report described that five inpatients with schizophrenia became irritable, uncooperative, and overactive with disturbed sleep after smoking ginseng-containing cigarettes and that, after cessation of smoking these cigarettes, these behavioural symptoms improved [34].

### 3.2. *Ephedra sinica*

*Ephedra sinica* (ma-huang) is an evergreen shrub native to Central Asia, whose dried parts have been used for thousands of years in the traditional Chinese medicine for the treatment of various respiratory conditions [35]. Its main biologically active component is the alkaloid ephedrine, a sympathomimetic agent [36]. The chemical structure of ephedrine is very similar to that of amphetamine; in fact, amphetamines have been originally synthesized as a substitute for ephedrine [36].

In 1972, a Danish physician first noted unintentional weight loss in a patient taking ephedrine-containing pills for asthma [37]. Subsequently, ma-huang became one of the most commonly used herbs for weight loss [38]. A meta-analysis reported that ma-huang promoted modest short-term weight loss (approximately 0.6 kg/mo more than placebo), although there are no data regarding long-term outcomes [39]. This effect is said to be mainly due to a sympathomimetic stimulation of thermogenesis in skeletal muscles [37, 40]. Ephedrine can rapidly pass through the blood-brain barrier [35] and can stimulate short-term release of dopamine and norepinephrine, as well as long-term depletion of monoamine [41]. Ma-huang use has been associated with several psychiatric adverse effects (reviewed below).

A meta-analysis of 50 trials on the use of ma-huang in the treatment of obesity yielded estimates of 2.2- to 3.6-fold increases in odds of developing psychiatric symptoms including euphoria, neurotic behavior, agitation, depressed mood, giddiness, irritability, and anxiety [39]. In a review of 1,820 previously unpublished Food and Drug Administration (FDA) reports of adverse events concerning ma-huang, the use of the herb was associated with 57 serious psychiatric episodes [41]. Thirty-two (56.1%) of these cases included reports of psychosis [41]. Other common adverse events (not mutually exclusive) were severe depression (31.6%), mania or severe agitation (26.3%), hallucinations, sleep disturbance, and suicidal ideation (22.8% each) [41]. Of the 55 cases for which gender was reported, 60% were women [41]. Most patients (59.6%) had been using ephedra for more than 2 months [41]. Two-thirds of the 57 cases involved patients with preexisting psychological/psychiatric conditions and/or the use of other mood-altering medications or illicit substances [41].

The first two cases of psychosis (one case of paranoid psychosis and one case characterized by depression, paranoid features, and vivid auditory hallucinations) caused by ephedrine were described in 1968 by Herridge and a'Brook [42]. Both patients reportedly used ephedrine to treat respiratory diseases and had no previous mental illnesses [42]. A 1987 review of 20 cases of psychosis in patients mainly taking ephedrine for respiratory diseases indicated the following: (i) the typical clinical picture of ephedrine-related psychosis was very similar to amphetamine-related psychosis, that is, a paranoid psychosis with delusions and auditory hallucinations (less frequently visual hallucinations) in a setting of clear consciousness; (ii) in the majority of cases, psychiatric symptoms remitted following abstinence from ephedrine; (iii) there was no personal or family history of psychosis in 18 of the cases; (iv) 80% of the patients had taken ephedrine for more than 1 year; and (v) the average dose before the psychotic episode was 510 mg [36]. Among the described 20 patients, one patient suffered from “neurasthenia,” one from “panic attacks,” three from “abnormal premorbid personality,” and two from substance abuse (morphine, amphetamine, and LSD); all others had no history of previous psychiatric disturbances [42]. Additional cases of ephedrine-induced psychosis have more recently been described [43–46].

In relation to mood disorders, several published case reports reported the association between ma-huang use and manic-like symptoms, such as psychomotor agitation, pressured speech, flight of ideas, suicidal ideation, irritability, aggressive and disorganized behavior, and reduced need for sleep [45–50]. Manic-like symptoms occurred both in patients with no previous psychiatric illness and in patients with a family history of bipolar disorder. These manic-like symptoms occurred when ma-huang was being assumed at the manufacturer recommended dosages, at doses exceeding the recommended amounts, or after dose escalation (the exact dosages were not reported) [45–50]. Following acute manic episodes, symptom remission was typically achieved with or without pharmacotherapy [45–50], although one patient reportedly developed long-lasting residual disturbances such as boundary control and obtrusive, loud speech [47]. Two case reports also detected the association between ma-huang use and depressed mood [44, 45], with depressive symptoms occurring after 1 year of use [44] or after the first use [45] of the herb. Another case report described a woman with severe obsessive-compulsive disorder who was effectively treated with fluvoxamine and Eye Movement Desensitization and Reprocessing (EMDR) treatments but relapsed following the ingestion of a single capsule of an herbal product containing ma-huang used to facilitate weight loss [51].

An additional relevant adverse psychiatric effect possibly related to ma-huang use is the onset of ephedrine addiction. Miller and Waite reported the case of a 20-year-old male with no psychiatric comorbidity whose use of a dietary supplement containing ma-huang progressed to substance abuse and quite possibly substance dependence [52]. He progressively escalated the assumed dose to more than four times the recommended amount (one capsule, two times per day), exhibiting symptoms of tolerance and withdrawal [52]. According to the product label, each capsule purportedly contained 313 mg of ephedra extract (8% concentration, 25 mg of ephedrine group alkaloids) and 50 mg of anhydrous caffeine [52]. In a case series of seven patients with amphetamine-like abuse or dependence, two showed ephedrine abuse and three ephedrine dependence according to the DSM-IV diagnostic criteria [53]. The 2001 FDA reports of adverse events found five patients (8.6%) that either self-reported or were diagnosed as being addicted to ma-huang products [41]. Finally, a study conducted on 64 female weightlifters identified 36 ephedrine users, 7 of which (19%) showed frank ephedrine dependence, including tolerance and withdrawal symptoms, persistent use despite adverse effects, and multiple unsuccessful attempts to discontinue use [54].

### 3.3. *Paullinia cupana*

*Paullinia cupana* (guarana) is a plant native to the Amazon region and is especially common in Brazil [55]. Guarana seeds contain a wide number of stimulant and psychoactive substances, including twice the amount of caffeine present in coffee beans and other xanthine alkaloids such as theobromine and theophylline [55, 56]. The plant is commonly used in soft drinks and energy drinks but its stimulant properties have made it a frequent ingredient in weight-loss compounds, with some evidence supporting its weight-loss effectiveness [47–49].

Due to the high caffeine content of its seeds, excessive intake of guarana may cause symptoms of caffeine intoxication (e.g., nausea, palpitations, anxiety, excitement, insomnia, and irritability) [23, 57, 58]. Baghkhani and Jafari reported the case of a patient experiencing heart palpitations associated with anxiety and irritability after using herbal supplements containing guarana (with a daily dose of 400 mg to 4 g) [59]. The World Health Organization-Adverse Drug Reactions (WHO-ADR) database reported guarana to be associated with cases of personality disorder, manic reaction, insomnia, somnolence, asthenia, fatigue, anxiety, and impaired concentration [23, 60]. Galduróz and Carlini [61] and Boozer et al. [62] conducted clinical trials aimed at assessing the efficacy of herbal supplement containing guarana for cognitive enhancement [61] or weight loss [62] reporting insomnia among the adverse symptoms most frequently experienced by participants.

### 3.4. *Pausinystalia yohimbe*

*Pausinystalia yohimbe* is a plant native to Central Africa [63]. The association between yohimbine, an alkaloid derived from the bark of the plant, and weight loss has been consistently reported [23, 63–66]. For example, Kucio et al. [63] documented that, compared to placebo, yohimbine (5 mg taken 4 times a day for 3 weeks) combined with a low-energy diet significantly increased the mean weight loss in obese patients. Yohimbine is an alpha2-adrenoceptor antagonist that produces sympathetic activation by increasing noradrenaline release and the firing rate of neurons located in noradrenergic nuclei of the central nervous system (CNS) [67, 68]. Consistently, increases in anxiety symptoms are the most commonly reported adverse psychiatric effects associated with yohimbine, both in healthy people [69, 70] and in psychiatric patients (especially in those with preexistent anxiety disorders) [71–76].

In healthy individuals, an intravenous dose of yohimbine (0.15 mg/kg up to a maximum dose of 10 mg) produced a significant increase in anxiety symptoms (e.g., mental and physical anxiety and irritability), along with an increase in systolic and diastolic blood pressure, and hyperventilation [69]. A significant decrease of cerebral blood flow, especially in the cortical regions, following yohimbine administration has also been observed [69]. Charney et al. reported that, compared to placebo, an intravenous dose of yohimbine (0.4 mg/kg) in patients with panic disorder was associated with a significant increase in anxiety ratings after 15 and 30 minutes [71]. A trend towards a significant increase in anxiety levels was also observed in healthy individuals after 15 minutes of yohimbine administration [71]. It has been reported [72, 73] that oral administration of yohimbine (20 mg) can produce a significant increase of subjective (i.e., nervousness and frequent panic attacks) and objective (i.e., palpitations, hot and cold flashes, tremors, etc.) anxiety symptoms in patients with agoraphobia. Furthermore, Gurguis et al. reported that the oral administration of yohimbine (20 mg) induced panic episodes in 6 of 11 patients diagnosed with agoraphobia with panic attacks and significantly increased systolic blood pressure, plasma norepinephrine, and cortisol levels [76]. The increase of anxiety symptoms after intravenous dose of yohimbine (0.4 mg/kg) has been also documented in combat veterans with posttraumatic stress disorder (PTSD) [74, 75], who concomitantly showed significant decreases in brain metabolism after yohimbine administration compared to healthy controls [75].

In addition to anxiety, yohimbine has also been associated with other adverse psychiatric effects. Yohimbine has been reported to induce addictive drug seeking behaviour [77], objective and subjective withdrawal, and elevated craving in heroin-dependent patients [78]. Scherr et al. [79] described the case of a 21-year-old female patient who developed alterations in alcohol and cocaine seeking behaviour and several modifications in behaviour, including suicidal tendencies, following the use of yohimbine for weight loss. It is reported that, compared to placebo, oral administration of yohimbine (0.4 mg/kg [80] or up to 40 mg [81]) can increase impulsivity in healthy subjects [80, 81]. Finally, Price et al. [82] described three cases of patients with bipolar depression who developed manic symptoms after taking a 10 mg dose of yohimbine. The US National Institute of Health states that “*people with psychiatric conditions should not use Yohimbe*” [83].

## 4. Interaction between Weight-Loss-Related Herbal Products and Psychiatric Medications

Herb-drug interactions have become an important issue in drug safety and clinical practice, possibly resulting in drug toxicities, reduced pharmacological effects, and adverse drug reactions. In this section we review the available evidence of interactions between HWLPs and psychiatric medications.

### 4.1. *Panax ginseng*

Two case reports suggest an interaction between ginseng and phenelzine [84–86]. A 64-year-old woman described symptoms of headache and tremulousness when ginseng was added to her phenelzine therapy; three years later, whilst still taking phenelzine, she experienced similar symptoms upon ingesting ginseng capsules [85, 86]. A 43-year-old woman who had had a long-standing depressive illness and whose medication included phenelzine 45 mg/day, triazolam 0.5 mg/day, and lorazepam 4 mg/day experienced an improvement in her depression; this improvement escalated into manic-like symptoms whilst taking a combination of ginseng and bee pollen. When the ginseng preparation was discontinued, she no longer experienced any therapeutic benefit of the phenelzine [84]. An interaction between ginseng, vitamin B complex, and sertraline resulting in a reduction of therapeutic response has also been reported [24]. Ginseng contains a mixture of saponin glycosides called ginsenosides. It has been found that ginsenosides inhibit cyclic AMP phosphodiesterase [87]; this may account partly for ginseng interaction with monoamine oxidase inhibitors (MAOIs) such as phenelzine, although an exact delineation of the mechanism would require further study.

### 4.2. *Ephedra sinica*

As written above, the predominant active component of ma-huang, that is, ephedrine, is a sympathomimetic agent with a chemical structure very similar to that of amphetamine [36]. It acts directly and indirectly at alpha- and beta-adrenergic receptors, potentially causing increased blood pressure and heart rate, relaxation of bronchial and gastrointestinal smooth muscle, CNS stimulation, and mydriasis [88]. When ma-huang is administered in combination with MAOIs, its sympathomimetic activity can reportedly increase the risk of hypertensive crisis [89]. It is reported that a 28-year-old woman taking phenelzine (60 mg/day) developed encephalopathy, neuromuscular irritability, hypotension, tachycardia, rhabdomyolysis, and hyperthermia following ingestion of a combination tablet containing ephedrine (18.31 mg), caffeine (30 mg), and theophylline (100 mg), 24 hours after abruptly discontinuing phenelzine treatment [89]. However, it is also possible that stopping the phenelzine suddenly contributed to inducing the side effects.

### 4.3. *Paullinia cupana*

Guarana extracts have high content of caffeine [55, 56], which is metabolized by CYP1A2 (a protein of the cytochrome P450 family of isoenzymes responsible for the biotransformation of several drugs) [58, 90]. When used concomitantly, caffeine and certain psychiatric medications (e.g., clozapine [91–94] and fluvoxamine [92]) can compete for the same enzyme, resulting in potential increases in medications' drug levels and risks of toxic effects [91–94]. One study has also reported that abruptly ceasing the daily consumption of caffeine may result in a significant increase in lithium blood levels [95]. However, no studies have reported interactions between guarana and psychiatric medications so far.

### 4.4. *Pausinystalia yohimbe*

Pharmacodynamic and pharmacokinetic interactions between yohimbine and certain tricyclic antidepressants (i.e., clomipramine, desmethylclomipramine, and imipramine) have been described [96–98]. It has been reported that yohimbine toxicity is potentiated by tricyclic antidepressants and phenothiazines in mice [99]. Lacomblez et al. [97] performed a double-blind, crossover, placebo-controlled study in 12 depressed patients with clomipramine-induced orthostatic hypotension reporting that low oral doses (4 mg three times a day) of yohimbine increased blood pressure, subsequently proposing yohimbine as a possible drug-induced orthostatic hypotension corrector. In addition, one preclinical [100] and one clinical study [90] suggested that concomitant administration of yohimbine may lead to more rapid rates of psychiatric effects of selective serotonin reuptake inhibitors (SSRIs) by shortening the delay in obtaining tonic activation of the postsynaptic 5-HT1a receptors through its alpha2-antagonist activity. The US National Institute of Health claimed that “*people should not combine Yohimbe with monoamine oxidase inhibitors as effects may be additive. Yohimbe should be used with caution when taken with medicines for high blood pressure, tricyclic antidepressants, or phenothiazines*” [83].

### 4.5. *Plantago psyllium*

Fibre supplements of* Plantago psyllium* (psyllium) may contribute to a salutary lipid, glucose, and insulin metabolism and are extensively used for weight-loss purposes [101]. Two studies to date have reported interactions between psyllium and psychiatric drugs: Fong et al. recently found that psyllium supplements significantly altered blood concentrations of carbamazepine [102], while Perlman in 1990 reported that psyllium can inhibit the absorption of lithium from the gastrointestinal tract leading to reduced blood concentrations and clinical effects of the drug [103].

### 4.6. *Rhodiola rosea*

*Rhodiola rosea* (*Rhodiola*) has a long history as a valuable medicinal plant having appeared in the Materia Medica of a number of European countries [104]. As a dietary supplement, numerous preparations of* Rhodiola* extracts are used worldwide as “adaptogen” substances [105].* Rhodiola* use has been suggested to reduce food intake [47, 48] and to inhibit angiotensin converting enzyme (ACE), resulting in a possible positive effect on metabolism [106].* Rhodiola* can modulate hypothalamic-pituitary-adrenal (HPA) axis activity as well as that of several neurotransmitters [104]. Maniscalco et al. recently reported the case of a 68-year-old female patient with recurrent moderate depressive disorder with somatic syndrome who developed vegetative syndrome, feelings of restlessness, and trembling since she began ingesting* Rhodiola* supplements in addition to paroxetine [107]. These symptoms may have been due to serotonergic syndrome [107]; therefore, it is possible that concomitant use of* Rhodiola* and selective SSRIs may lead to an excess of serotonin in the CNS and/or in peripheral nervous system with subsequent increased clinical risks.

### 4.7. *Trigonella foenum-graecum*

Seed extracts of* Trigonella foenum-graecum* (fenugreek) have long been used as an herbal medicine for treating metabolic and nutritive dysfunctions and have been shown to selectively reduce spontaneous fat intake in overweight subjects [108]. Pharmacodynamic interactions of fenugreek with MAOIs and SSRIs have been described* in vitro* [109], although reliable human data are lacking.

## 5. Conclusion

The consumption of HWLPs as the fastest and “healthiest” way to lose weight is a relatively new and poorly studied phenomenon. To the best of our knowledge, this is the first study aimed at specifically reviewing the adverse psychiatric effects associated with HWLPs. Results indicate that a range of adverse psychiatric events could theoretically result from the use of HWLPs. Though the evidence of psychiatric symptoms associated with HWLPs may collectively look impressive, the information provided in the reviewed studies is often insufficient for making reliable inferences about causality; therefore, in some cases it may be unnecessarily alarmist to accept a cause-effect relationship between HWLPs and psychiatric symptoms.

In addition to the reviewed evidence (i.e., the effects of HWLPs* per se* on psychiatric symptoms and the interactions of HWLPs with psychiatric drugs), there are four further reasons of possible concern in relation to the potential psychiatric adverse effects of certain HWLPs.

(i) The HWLPs may contain adulterants with the potential to cause neurotoxicity or adverse interactions. Herbal products, in fact, undergo reduced quality control and safety testing in comparison to medications [18]. For example, adulterants that have been identified in HWLPs include sibutramine [110], fenfluramine [111], or thyroid hormones [112, 113], which are molecules with well-known potential deleterious effects on CNS, mental status, and behaviour [114–116]. (ii) Individuals are using increasingly large amounts of herbal products as “weight-loss adjuvant interventions” in addition to the standard HWLPs. For example,* Valeriana officinalis* (valerian),* Passiflora incarnata* (purple passionflower),* Melissa officinalis* (lemon balm), and* Hypericum perforatum* (St. John's wort) have long been used to improve sleep, anxiety, or mood disturbances [117–119]; these herbal products can interact with a range of neurotransmitter systems, having the potential to induce adverse psychiatric and cognitive effects [112, 113, 118, 120–124] and to interact with psychiatric medications [118, 125–127]. Even though extracts of these herbs have never been shown to improve obesity or metabolism, they are often sold on the Internet with the purpose of calming stress-induced hunger and eating behaviours related to nervousness [19, 128–130]. People, in fact, are becoming increasingly aware of the link between mood, poor sleep quality, and obesity [19, 128, 130, 131]. In an attempt to address mood and sleep disturbances, consumers may use products containing extracts of these plants, considering this intervention to be a relevant part of their weight-loss program [19, 128–130] and subsequently increasing their risk of adverse psychiatric effects. (iii) A range of HWLPs can interact with enzymes involved in psychiatric drug metabolism [132], having the potential to interfere with pharmacokinetic mechanisms. (iv) Finally, obesity is associated with significant increases in lifetime diagnosis of mood and anxiety disorders [133, 134]. It is possible that obesity* per se* may lead to higher vulnerability to the development of psychiatric disorders [133], meaning that obese individuals may represent a psychiatrically at-risk population and the use of HWLPs with potential adverse psychiatric effects among them may be of particular concern.

In conclusion, adverse psychiatric events and interactions with psychiatric drugs are reported for a number of herbal products commonly used for reducing body weight. Physicians, psychiatrists, and other health and mental health professionals should remind patients that (i) diet, exercise, and healthy lifestyle are the safest and most effective weight-loss strategies and (ii) the HWLPs, though potentially effective for weight loss, may have a heterogeneous nature and may be associated with psychiatric risks. It is also true that certain HWLPs may exert beneficial effects on CNS and on certain psychiatric conditions [135], but this analysis was not included as a part of the objective of the present review. Future studies should include safety mental health measures in relation to this growing pattern of substance use.
